# Protection of Location Privacy Based on Distributed Collaborative Recommendations

**DOI:** 10.1371/journal.pone.0163053

**Published:** 2016-09-20

**Authors:** Peng Wang, Jing Yang, Jian-Pei Zhang

**Affiliations:** 1 College of Computer Science and Technology, Harbin Engineering University, Heilongjiang, China; 2 College of Information Engineering, Suihua University, Heilongjiang, China; West Virginia University, UNITED STATES

## Abstract

In the existing centralized location services system structure, the server is easily attracted and be the communication bottleneck. It caused the disclosure of users’ location. For this, we presented a new distributed collaborative recommendation strategy that is based on the distributed system. In this strategy, each node establishes profiles of their own location information. When requests for location services appear, the user can obtain the corresponding location services according to the recommendation of the neighboring users’ location information profiles. If no suitable recommended location service results are obtained, then the user can send a service request to the server according to the construction of a *k*-anonymous data set with a centroid position of the neighbors. In this strategy, we designed a new model of distributed collaborative recommendation location service based on the users’ location information profiles and used generalization and encryption to ensure the safety of the user’s location information privacy. Finally, we used the real location data set to make theoretical and experimental analysis. And the results show that the strategy proposed in this paper is capable of reducing the frequency of access to the location server, providing better location services and protecting better the user’s location privacy.

## Introduction

Location-based services (LBS) provide a variety of information services based on the location coordinates of the user [1]. In recent years, with the rapid development of mobile terminals, a variety of mobile applications have been widely used. At the same time, with the development of positioning technology, an application can obtain location information anytime and anywhere and provide location-based services for users. Under the existing location-based service framework, users use their mobile phones and other smart devices to send their location information and query requests to an LBS server, and then the LBS server returns the location-based service according to the user’s location. General LBS applications include interest point queries and route navigation, such as querying the nearest bank and seeking the nearest waiting site, etc. While this service brings convenience for users, the disclosure of user’s location becomes the main concerns. The location information leak will be a large threat to the user's personal privacy. Crimes could also occur occasionally because of a location privacy leak. Thus, the problem of location privacy protection must be solved for LBS applications.

Gruteser and Grunwald [2] firstly noted that using the k-anonymous privacy model of the database [3] can be applied to location privacy protection. Chow [4] and his partners summarized a location privacy protection scheme in LBS from the aspect of system structure. Most of the existing location privacy protection schemes use a centralized structure with a trusted anonymous server, and the user's precise location is generalized to meet the demands of the area that it is in. Obviously, the centralized structure of the trusted anonymous server will become the communication bottleneck and the point to be attacked.

Chow presented space anonymous methods based on the Peer-to-Peer structure [5], but this method mainly solves the problem of how to use the neighboring node location information to implement the *k*-anonymous privacy protection method in a distributed system, and the authors assumed that it is credible among the nodes, and thus, they ignored the problem of privacy protection among neighboring nodes. In the literature, Reza [6] proposed using LBS services that are nearby to the users as a sharing mechanism to reduce the chance of the users’ own positions being exposed to the server. The node sets a buffer to store the users’ previous location-based services from the LBS server in their paper, which is used to provide location services for the neighboring users later. However, this approach faces a cold start, and the initial LBS services request privacy issues. According to the above problems, this paper proposed using a collaborative recommendation algorithm that is based on the distributed structure in the literature [5], it uses the neighboring user's location information to provide location services recommendations, and it considers the location privacy protection problem among the neighboring nodes. Additionally, the data transmission between two neighboring nodes adopts Paillier homomorphic encryption methods to enhance the user’s location privacy. We take full advantage of the features of the distributed system, decentralize the recommendation service computing tasks to the neighboring nodes, and effectively solve the problem of key-nodes under a heavy load in the existing scheme.

Our main contributions can be summarized as follows:

1. A novel model of distributed collaborative recommendation location services strategy (DCRLS) is proposed by using the neighboring users’ location information profiles to recommend location service. It aims to decrease the frequency of access to the location server and reduce the risk of privacy leak from the suspect severs.
2. Using generalization to process location information profiles and using centroid instead of the user’s real location to prevent attack. And we encrypted the user’s location information profile by adopting Paillier cryptosystem to protect the user’s location information.
3. We verified the feasibility and effectiveness of the algorithm by using real data sets and compared performance indicators of data utility and communication cost with the existing location privacy protect methods. Empirical studies suggest that our location privacy model is better to get location service result sets, at the same time, it can reduce the frequency of access to LBS server and protect better the user’s location privacy.

The rest of the paper is organized as follows. Section 2 presents overview of related work. Section 3 illustrates the steps of DCRLS’s work process. Section 4 analyzes feasibility and performance of algorithm that proposed in this paper. Finally, Section 5 concludes and identifies research directions.

## Related Work

The largest location-based services (LBS) privacy threat has a great impact on users with time and space reasoning attacks because of the user's location information leakage. Currently, a large amount of research has published summaries about LBS privacy protection [7, 8]. For privacy protection based on LBS, the main aim is to use privacy protection technology that makes the attackers unable to obtain any information about the precise location of the users under the LBS services, which can be applied under normal circumstances. The main methods can be divided into regional coverage, deception of location, data encryption and other methods [9].

Regional covering technology is the most common method for protecting the location information [10–12]; its main idea is to use a larger area to take the place of the original point to make others unable to obtain the precise location of the target. Additionally, it is mostly a method that is based on protection using *k*-anonymous [13]. Each user anonymously uploads an area instead of the original precise location, and the area contains at least k users. When the attackers obtain the information about the area of the users, they cannot distinguish the user making the current request from a set of k users. The existing literature [14] uses the users’ location information to achieve a k-anonymous area through the AD hoc network that is formed among the users. When users need location services, users query the number and position of the neighboring users by neighboring radio signals before sending a request to the server. If the result does not meet the condition of anonymity, the neighbor continues to broadcast the request until it finds more than *k* users, and according to the users’ location, the coverage area is generated. If the area is smaller than the smallest area for privacy regulations, it must expand. Then, the users use the generated area instead of their own position to achieve the protection of the users’ location privacy. Compared with k-anonymous algorithm, differential mechanism of privacy is widely applied in the field of privacy protecting because of its solid theoretical basis, such as differential mechanism of privacy is applied in BANS [15,16].

Cryptography has been widely applied in the location privacy problem and makes the server unable to obtain the users’ location information through an encryption or other mathematical transformation [17–19]. Compared with the previous two types of location privacy protection technology, cryptography technology is more thorough and safer for location privacy protection. It completely eliminates the attacker's threat in theory, but the shortcoming of the cryptography methods is high computational complexity.

In addition to the above three types of main methods for the location privacy protection, there are also some other location privacy protection methods that are based on the LBS system structure [20–25]. For example, there is literature [20] on using a cache in a distributed system. When there is a request for LBSs, a user can query the local cache from the cache data on the local cell phone and not expose the location information to the server. There is literature [21] that uses a combination of a cache with user collaboration for location privacy protection, and the purpose is to avoid the user sending a request to the server directly. Each user has a cache of their recently requested results. The literature [22] uses a P2P structure to protect the location privacy, which achieves the protection of privacy by the abilities of the mobile devices themselves in addition to coordination. Compared with the existing centralization location service structure, the system structure based on distributed location service has great advantage. For example, an anonymous space method based on Peer-to-Peer is proposed [5]. This method uses location information of neighboring nodes to establish the k anonymous privacy protect method, but it ignores the location privacy protecting among the neighboring nodes. LBS shared mechanism of neighboring users is proposed [6] to reduce the exposure of location. It sets a buffer to store its own previous LBS service from the server, but it faces the cold start of location services.

In this paper, we introduced a user profile model that is based on a distributed system structure, a novel distributed recommend location service model based on user information profile. Compared with existing distributed location service, advantages of the scheme proposed in this paper are as follows. (1)We adopted user location information profile and used data set of user location instead of one location as the object. The scheme has fully considerations about the privacy problem among adjacent groups and uses the method of encryption to transmit the location data; (2) Although the strategy adopts the k-anonymity scheme as the alternative after users cannot get the recommend location service, but it uses the k-anonymity data set instead of real location, so this strategy can protect privacy better. (3) In this paper, the dependency of the proposed scheme on the LBS server is enough lower to reduce the frequency of access to the server and overcomes the cold start problem of the LBS services at the same time.

## Methods

### Summary description of the scheme

**Fig 1:**
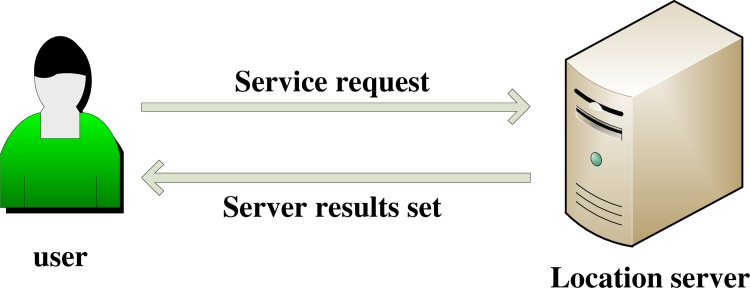
Central server mode.

Existing LBS service system architectures usually adopt a centralized server structure in which the users send their own locations and query services to the server, and then, the server returns the LBS services back to the users according to the location information, as shown in Fig 1. In this mode, if the server system has security holes or the staff intends to reveal information, the users’ location information will be leaked.

**Fig 2:**
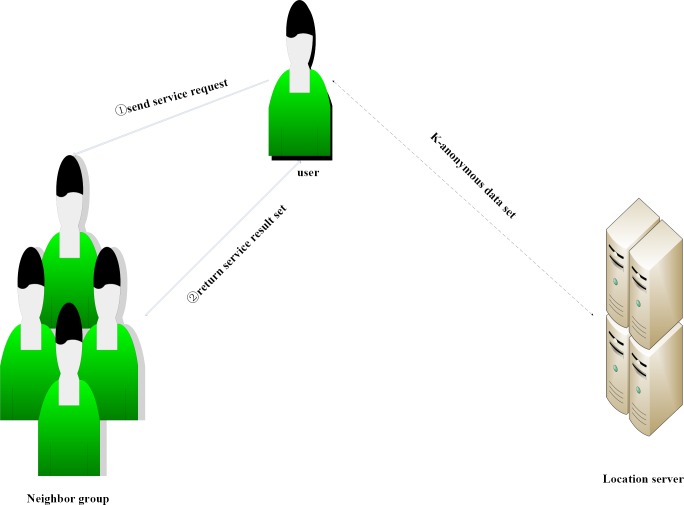
Collaborative recommendation service mode.

In this scheme, each user collects their location information to construct location information profile. When a user needs to recommend location services, the user makes a request to the neighbors, and then, the neighboring users recommend the LBS service results, and the user requires secondary screening by the neighboring users’ LBS service data sets to obtain satisfactory position service data. If there is no satisfactory result on the recommended location services, then the user sends a service request to the server by using the neighboring users’ centroid position information to structure the *k*-anonymous data set. This process is shown in Fig 2.

The strategy recommends location-based services for users in a distributed system structure based on the collaborative recommendation algorithm, and it overcomes the communication bottleneck and the defect with respect to the anonymous centralized system structure; it does not need to change the existing LBS structure at the same time and introduce a third party platform to reduce the cost of the system. It is necessary to explain that the collaborative recommendation method raised in this paper is different from the traditional collaborative filtering algorithm. The traditional collaborative filtering algorithm is a more popular technology for recommending commodities for users by the analysis of the historical data of other user groups and technologies [24]. Instead, the method in this paper uses the location information profile of the user group data to recommend the location information, and thus, it uses encryption appropriately, while accounting for the users’ location information privacy and the balance of the computational load on the problems that involve the position profile measurement. The algorithm guarantees information privacy for the users.

### Structure location profile

In our work, each node collects its location information form a profile. The profile information is generated by the node’s location information and stored in the memory of the node. Thus, there is no privacy leak in the process of the profile information that is generated. When we receive the LBS request from the other users, the user uses his location information profile to recommend suitable location result sets, and thus, the location information profile of the node determines the quality of the recommend LBS services results. The method in this paper considers fully the dwell time, access frequency, tag parameters and so on, with regard to the structure of the node location data profile.

In this scheme, each user node is expressed by the user's location information profile, and it is assumed that the location information profile is a set that is constituted by the position information of *k* users. Each element in the set can be represented as a (*L*(*x*,*y*),*l*,*t*,*n*), where L(*x*,*y*) is the position of the user, (*x*,*y*) is the coordinates of the position, *l* is the topic labels of the position, such as hospitals, banks, shops, *t* is the dwell time of the user at the position, and *n* is the number of times the users visit the position. For richness and usability of the users’ location information profile, this paper makes full consideration of the dwell time, access frequency, tag parameters and so on, and we ranked and filtered the data in the position information set by the location profile generation algorithm and generated a dynamic location information profile of the users.

User *Ua* uses an intelligent mobile terminal to collect the location information point according to certain rules, for example, the dwell time at a certain position is longer than the default threshold of the dwell time; then, the position will be added to profile set A, the set can store k position elements, and thus, we can obtain the initial set of the users’ location information, as show in formula (1).

$$A=\left\{\left(L^{1},l^{1},t^{1},n^{1}\right),\left(L^{2},l^{2},t^{2},n^{2}\right),\left(L^{3},l^{3},t^{3},n^{3}\right)\ldots \left(L^{k},l^{k},t^{k},n^{k}\right)\right\}$$ (1)

When a new location point meets the threshold for the dwell time, we used the dynamic profile generation algorithm to adjust the outline of the user's location information; then, we determined the new user's location information profile and calculate the centroid position while considering the dwell time and visit time.

The design of the algorithm is as follows:

**Algorithm 1** Location information profile generation

Input: empty set *A*, threshold of dwell time Δ*t*, new dwell position *x*

Output: A'

1: If *x*.*t*≥Δ*t*

2: for all *a*∈*A* do

3: if *a*.*L* = = *x*.*L* //whether position x exists

4: Update the position *a* information;

5: if *A*.length<*k*;

6: insert *x* to *A*;

7: dynamically adjust the set according to the access time

8: return *A'*

**Fig 3:**
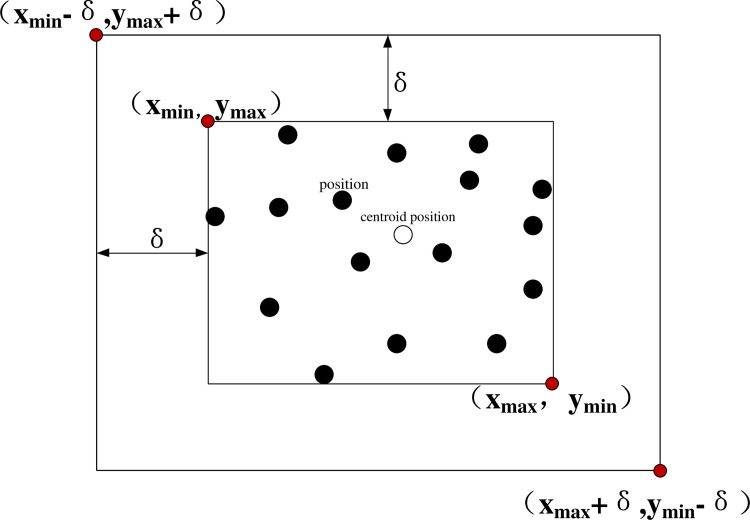
Profile of the node position.

For position *x*, Steps 1–4 update the existing position in the location profile set, and Steps 5–7 dynamically adjust the elements of location profile set. The user's location information profile *A* as generated by algorithm 1 can describe the location of the user better. It proves that the position point of the location information profile has certain cluster properties in the experimental part, such as family, work unit, shopping places, favorite restaurants, and so on. At last, the location profile of the user *Ua* can be described as a rectangular area that contains *k* positions; it uses {(*x*_min_ − *δ*, *y*_max_ + *δ*),(*x*_max_ + *δ*, *y*_max_ + *δ*)} to mark the location profile, where *X*_min_ is the minimum *x* coordinate in all elements of the location profile, and *X*_max_ is the maximum *x* coordinate in all of the elements of the location profile. Similarly, *Y*_min_ is the minimum *y* coordinate among all of the elements of the location profile, *Y*_max_ is the maximum *y* coordinate among all of the elements of the location profile, and *δ* is the offset. Here, (*x*_min_ − *δ*, *y*_max_ + *δ*) is the coordinate on the top left corner of the rectangle, and (*x*_max_ + *δ*, *y*_min_ − *δ*) is the coordinate on the lower right corner of the rectangle. Moreover, this approach defines the centroid position of the profile, which is (*avg*(*x*_1_,*x*_2_…*x*_*k*_),*avg*(*y*_1_,*y*_2_…*y*_*k*_)), as shown in Fig 3.

### Process of requesting location-based services

In this paper, it is assumed that user *A* requests location-based services at position *L*; then, the user broadcasts the request into the surrounding area in the designed scenario. The service information includes the location and the service request content. While considering the user's location information privacy, the strategy proposed in this paper generalizes the location information of user *A* to form a rectangular area *L*′ that hides the position *L*, and then, it uses the Paillier homomorphic encryption algorithm [26] to encrypt the information in the location service request. At last, the service request information can be formed into the following: *R* = (*pk*, *E*(*pk*, *L*′), *E*(*pk*, *r*), *Q*(*1*)), where *pk* is the public key generated by using the secret key to generate the algorithm, *E*(*pk*, *L'*) is the encrypted identification of the location information, *E*(*pk*,*r*) is the encrypted area offset, and *Q*(*l*) is the content about the theme tag *l* of the request queries.

**Fig 4:**
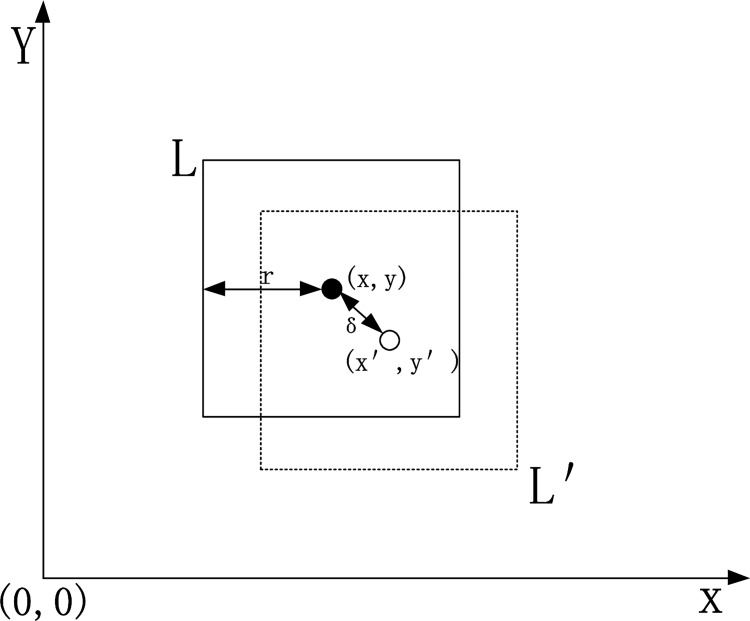
Position generalization.

It is assumed that the user's location *L* is (*x*, *y*). To avoid the location information being directly exposed to the other users at adjacent nodes, we generalize the *L* in this algorithm, which means that we set up a rectangular area that contains the user’s position, and then, it sends the rectangular area to the other users at adjacent nodes to reduce the chance of location leakage. Thus, it sets a variable *r* to measure the generalization uses (*x*, *y*) as the central point of the circle and uses *r* as the radius to generate a rectangle with an inscribed circle. The coordinate on the top left corner of (*x*-*r*, *y*+*r*) and the coordinate on the lower right corner is (*x*+*r*, *y*-*r*) in this area. To prevent center attacks, it generates randomly the offset *δ*, 0<*δ*<*r*/2, finally it forms the user's location rectangle, which is represented as *L*', as shown in the dashed rectangle of Fig 4.

As described above, when the user requests location-based services, the user’s location information is generalized into a rectangular area that contains the user’s position. However, if the coordinate information of the rectangular area broadcasts directly to the neighboring nodes, then there is a threat of location information leakage. Thus, the strategy proposed in this paper encrypts the user's location information by the Paillier password system.

(1) Initialization of a secret key

We randomly generate two large prime numbers, *p* and *q*, and count *n* = *p* * *q* to make gcd(*n*, *φ*(*n*)) = 1, when p = 2*p*′ + 1, q = 2q′ + 1, *m* = *p*′ * q′. Then, we randomly select the parameters $(a,b)\in Z_{n}^{*}\times$, $\beta \in Z_{n}^{*}$, and count *g* = (1 + *n*)^*a*^ * *b*^*n*^ mod *n*^2^, we generate the public key *pk* = (*g*, *n*) and the private key: *sk* = *βm*.

(2) Scheme of homomorphic encryption

It is assumed that (*E*,*D*,*K*) is a homomorphic encryption scheme and that the previous key generation algorithm generates a public key(*pk*) and a private key(*sk*). Thus, Paillier adds homomorphic properties, which are the following: 1. the two cipher messages’ additive operation result is equal to the corresponding two plain messages’ additive operation result, namely, any two numbers that belong to $Z_{n}^{*}$ (a and b), the public key pk have *D*(*E*(*pk*, *a*).*E*(*pk*, *b*), *sk*) = *a* + *b*. 2. There exists *D*(*E*(*pk*, *a*)^*r*^, *sk*) = *r*.*a* for the r power operation of the plain message *r*.*a* and for all *r* that belong to $Z_{n}^{*}$.

The algorithm in this paper uses the Paillier encryption scheme to encrypt the location information while considering its features to be suitable for the strategy. When user A demands an LBS service, the user generates a public key and a private key (*pk*, *sk*) by running the key generation algorithm for the encryption scheme (*E*, *D*, *K*); then, the user generalizes its position coordinate *L*' and finally sends the information *R* of the service request to the adjacent nodes.

### Response process of neighboring nodes

When the neighbor user *B* receives the information *R* of the service request from user *A*, user *B* uses *pk* to encrypt his profile *L_b_*, which is denoted as *E*(*pk*, *L_b_*), and obtains the encrypted location profile *L_b_*'. We set the profile encryption arithmetic *E*(*pk*, *L*') results of the user *A*’s position as *L_a_*'. Then, user *B* calculates the intersection of *L_b_*' and *L_a_*'. If there is no intersection, then user *B* does not respond to the service request information. In contrast, if there is an intersection, user *B* recommends location-based services to user *A*.

Definition 1: User’s location profile. The position of the neighboring user *B* is expressed as (*x_i_*,*y_i_*)(1≤*i*≤*k*), and thus, the corresponding location profile *L_b_* is as follows:

$$\{\begin{array}{c} x_{\min}=\min(x_{1},x_{2}\cdots x_{k})\\ x_{\max}=\max(x_{1},x_{2}\cdots x_{k})\\ y_{\min}=\min(y_{1},y_{2}\cdots y_{k})\\ y_{\max}=\max(y_{1},y_{2}\cdots y_{k})\\ L_{b}=\{(x_{\min},y_{\max}),(x_{\max},y_{\min})\} \end{array}$$ (2)

Here, (*x_min_*,*y_max_*) is the coordinate on the top left corner of the profile rectangle, and (*x_max_*,*y_min_*) is the coordinate on the bottom right corner of the profile rectangle. According to the received public key *pk* and the increment parameter *r*, user *B* calculates and encrypts their profiles as follows:

$$\{\begin{array}{l} x_{bz}=E(pk,x_{\min}).(-E(pk,r))\\ y_{bz}=E(pk,y_{\max}).E(pk,r)\\ x_{br}=E(pk,x_{\max}).E(pk,r)\\ y_{br}=E(pk,y_{\min}).(-E(pk,r))\\ E(pk,L_{b})=\{(x_{bz},y_{bz}),(x_{br},y_{br})\} \end{array}$$ (3)

After this calculation, (*x_bz_*, *y_bz_*) is the coordinate on the top left corner of the profile rectangular area, and (*x_br_*, *y_br_*) is the coordinate at the bottom right corner of the profile rectangular area. The location area of the service request received from the user *A* is {(*x_az_*, *y_az_*), (*x_ar_*, *y_ar_*)}.

The arithmetic of the intersection of B’s location profile and *A*’s location profile is as follows.

**Algorithm 2** Judgment of the profile intersection

Input:L_b_, L_a_

Output:*W* and (*x_avg_,y_avg_*)

1:*X_jz_*←max(*x_bz_, x_az_*)

2:*Y_jz_*←min(*y_bz_,y_az_*)

3:*X_jr_*←min(*x_br_, x_ar_*)

4:*Y_jr_*←max(*y_br_,y_ar_*)

5: if (*X_jz_*> *X_jr_*) or (*Y_jz_*< *Y_jr_*)

6:     EXIT

7: *x_avg_*←avg(*x_1_,x_2_……x_k_*);

8: *y_avg_*←avg(*y_1_,y_2_……y_k_*);

9: form recommend set *W* suiting theme *1*

10: return *W* and (*x_avg_,y_avg_*)

**Fig 5:**
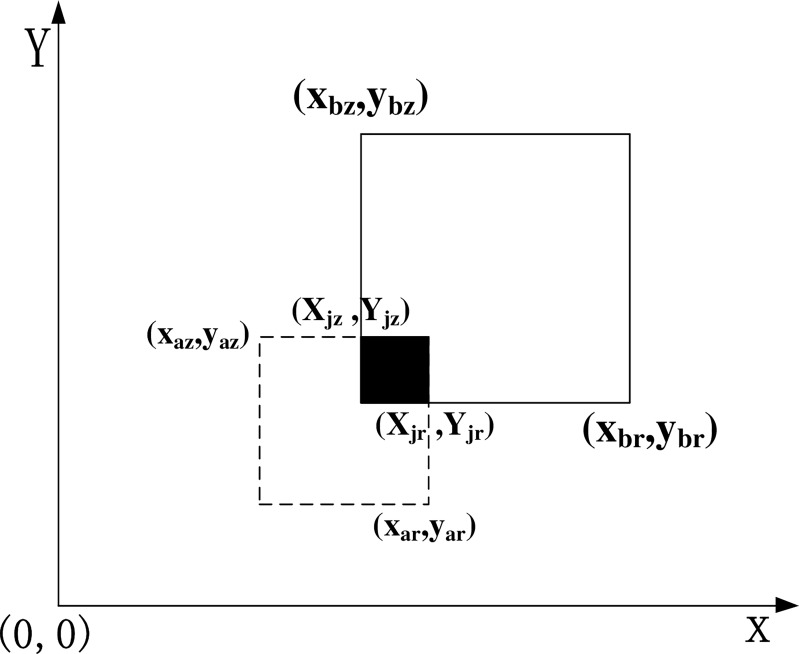
Intersection profile.

If there is an intersection of the two profile areas, the rectangular area is expressed as (*X_jz_*,*Y_jz_*),(*X_jr_*,*Y_jr_*), the (*X_jz_*,*Y_jz_*) is the coordinate on the top left corner of the rectangle, and the (*X_jr_*,*Y_jr_*) is the coordinate on the lower right of the rectangle. While *X_jz_*> *X_jr_* or *Y_jz_*< *Y_jr_*, there is no intersection of the two profile areas, as shown in Fig 5.

It is assumed that the location profile of the neighboring node *B* is labeled as *L_b_.* The response algorithm of node *B* is as follows: if there is an intersection of the two profiles, then user *B* searches the location information subject theme information in its own profile to form the recommended results set *W* and the centroid position. Then, the user B sends the positions of *W* and the centroid to user *A*.

### Sieving the recommendatory results data

It is assumed that user *A* obtains *m* neighboring users’ feedback sets of LBS services *W'* and the centroid position *L'*, where *W*’ = {*w*_1_, *w*_2_, …, *w*_*m*_}, *L*’ = {*l*_1_, *l*_2_, …, *l*_*m*_}. User *A* decrypts the set *W'* and *L'* in the first place and then screens the decrypted result set *W* to generate the candidate set *Q* or *k*-anonymity position set *L*, for which the algorithm is as follows:

**Algorithm 3** Sieving of the recommendatory results data

Input:LBS service result set *W’*, centroid position set *L’*

Output:candidate set *Q* or *k*-anonymity position set *L*

1: *W*←D(*W’*, *sk*) 

2: *L*←D(*L’*, *sk*) 

3: for (*i* = 1:*m*)

4:     for (*j* = 1: Q.length)

5:         Classify the elements in the *W* according to the residence time, visit times, recommended times, and deposit selective elements into the *Q*

6: Sort(*Q*)

7: if *Q*! = NULL

8:     return *Q*

9: else

10:     return *L*

Finally, we obtain the LBS result set *Q*; elements in this set are represented as (*L*(*x*, *y*), *L*, *t*, *n*, *count*), where *L*(*x*, *y*) is the coordinates, *l* is the theme label, *t* is the total residence time of the neighboring user at the location, *n* is the total number of times that the neighboring users access the location, and count is the number of times that the neighboring users are recommended. In this paper, the recommendation time is a priority measure; it can be changed according to the actual needs of the user, for example, the nearest distance or other measure. If the user is not satisfied with the results of the service, then the users can structure *k*-anonymity by using the centroid position information *L* to send LBS service requests to the server [5].

## Results and Discussion

In the experiments, we used a data set of cab mobility traces [27], and the data set contained 536 objects; each object contained GSP location information of the taxi over a month. There are a lot of research contents [28–31] based on transportation data sets, because the object has great advantage of real-time and data volume. In this section, we validated and analyzed the feature of the proposed scheme gradually according to the profile generation algorithm, LBS service request and response algorithm. The evaluation index mainly includes the generalized distance of the profile, the number of responding users, the number of service results and the success rate of the recommended services. We used the above-described aspects of the data to validate and analyze the data availability, the service quality service and the algorithm efficiency, which are the three aspects proposed in this paper. And then, we performed a comparison experiment among the DCRLS, P2P [5] and MobiCrow [6] in communication cost and performance aspects.

### Performance of the profile-generation algorithm

In this scenario, each node collects its positions, staying for a longer time to generate its own location profile. We use the location data profile to recommend the location services for the neighboring users when the LBS requests appear. Because the location information set of the node location profile has a large influence on the quality of the LBS service recommendation results, it requires full validation and analysis of the algorithm that is generated by the location profile of the real data set. In this process, there is full consideration of the number of nodes at the same time, the dwell time threshold of the node location area, the times of access, the theme tag and other variables.

The adopted data set contains location information of 536 taxis over a month; this location information is described by the latitude and longitude and also contains the time stamp that corresponds to the point position. The node location information profile generation algorithm attempts to screen the location of each object according to a certain priority, for example, the screening experiments on the dwell time parameters generate the position profile of the object. To determine the object positions, we counted the average speed between the two adjacent time stamps *v* (v=ΔsΔt), and then, we used *v* to measure the time that the objects stay in the corresponding areas. Obviously, when *v* = 0, the object is still in the period of time. Considering the special properties of using the taxi as the data set objects, we only consider to collect the still positions because the experiment outlines information points that form the corresponding location information outline. In the experiment, we processed the location information profile of the objects, 536 taxis by using a contour generation algorithm, then, we validated and analyzed the data. First, we selected at random 6 objects from 536 taxis, and finally, all of the taxi objects data is given.

**Fig 6:**
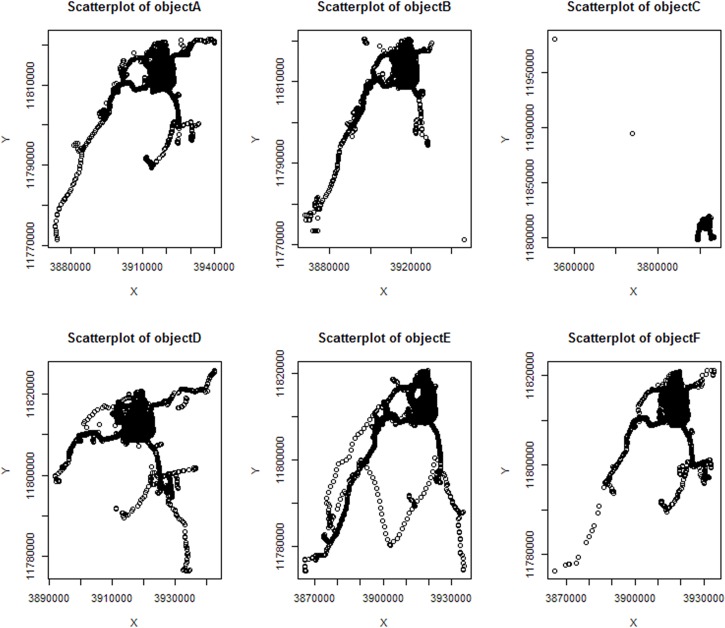
Scatterplots of the original location.

We selected at random 6 taxi objects, *A* ~ *F*, and the scatterplots of the original location data are shown in Fig 6.

**Fig 7:**
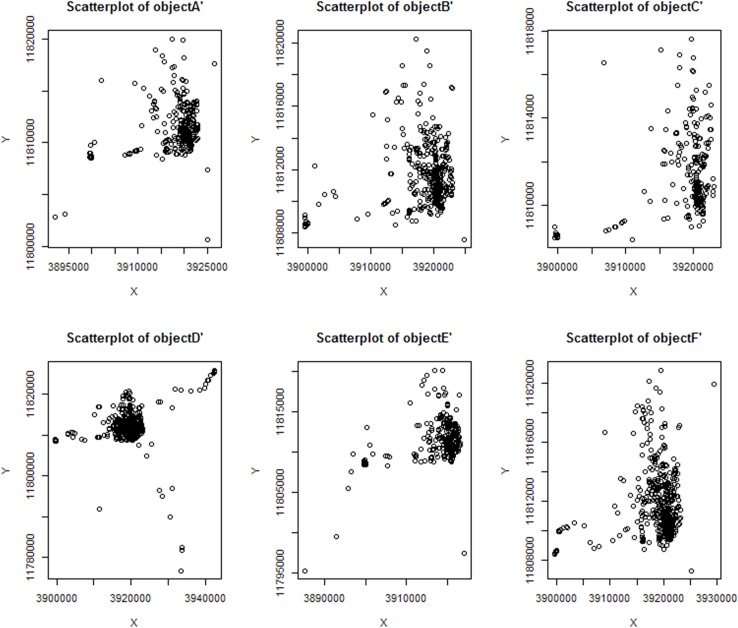
Scatterplots of the location information profile.

The scatterplot diagram of the corresponding location information profile generated by using the profile generation algorithm is shown in Fig 7.

In Figs 6 and 7, x-axis and y-axis represent the geographic coordinates of position. Compared with the position information changes of the six moving objects, the scatterplot shows that the generated object position outline identifies the activity area of the objects better. Additionally, the elements of the object profile have certain clustering. To further analyze the availability of the profile generation algorithm proposed in this paper, we counted the data of six moving objects, including the original location information, the location profile information rectangular area, the number of positions in this rectangle, which is denoted by the *numberOL*, the number of positions in this profile, which is denoted by the *numberLP*, the area ratio of the original location and the location profile, which is denoted by *ratioA*, and the data number ratio of the original location and location profile, which is denoted by *rationN*. These data are shown in Table 1.

**Table 1:** Data in the object profiles.

Name	Original location	Location profile	*numberOL*	*numberLP*	*ratioA*	*ratioN*
****objectA****	(3873626,11821482) (3940082,11771571)	(3892056,11820049) (3926633,11800674)	20543	596	20.2%	2.9%
****objectB****	(3867798,11820553)(3946396,11771350)	(3899564,11820254)(3924962,11807614)	20159	818	8.3%	4.06%
****objectC****	(3553922,11980549)(3933677,11798774)	(3899557,11817670)(3923071,11808405)	11616	354	1.34%	3.05%
****objectD****	(3891930,11825963)(3942674,11776498)	(3899573,11825953)(3942598,11776727)	22694	840	84.38%	3.7%
****objectE****	(3865557,11820873)(3935831,11774437)	(3885050,11820226)(3924267,11795321)	25611	978	29.93%	3.82%
****objectF****	(3864940,11821191)(3934646,11776343)	(3899619,11820901)(3929495,11807286)	26165	722	12.51%	2.76%

The results show that the point number ratio of the original location and the location profile generated by the location information profile generation algorithm is approximately 3%, from Table 1. This finding means that the profile generation algorithm that is proposed in this paper uses approximately 3% of the original positions in the data set collection form the position profile of the moving object. The area ratio of the node profile is associated with the movement area intensity of the moving objects, and the area ratio of node objectD is 84.38%. Compared with the two scatterplot diagrams of the original location data and the location profile data, it is obvious to find that the location profile points represent the original location information better. The area ratio of the node objectC is 1.34%, and the main reason is that the data from the original position of the moving objects has two outlier data points. Combined with the data in Table 1 and the node location information outline scatterplot, it is not difficult to find that the location information profile algorithm proposed in this paper can reject the outliers that has a short residence time and less access time to ensure a high quality utilization rate of the position data in the generated location information profiles. In addition, the location data in the generated nodes’ location information profile is sufficiently decreased to improve the efficiency of the data operation and storage.

**Fig 8:**
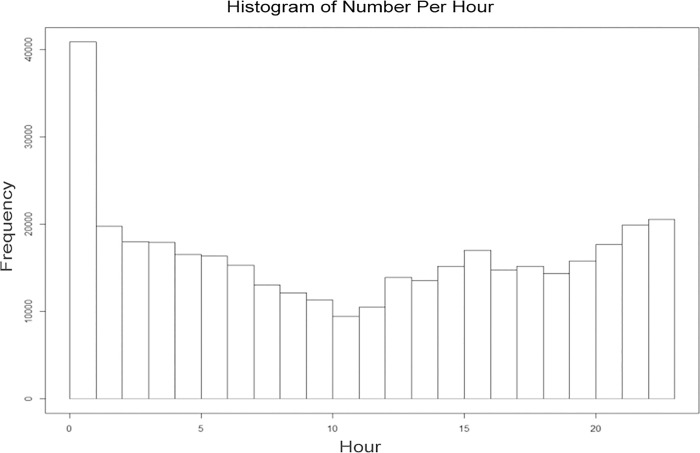
Location information quantity at different times.

We used the algorithm proposed in this paper to process all of the location information of the moving objects in the data set, and the final data statistics are as follows: the data set has 536 moving objects and a total of 11219955 location points, and thus, the average of the location points is 20930. There is a total of 378620 points; the average number of location points is 706, and the average rate of the points is 3.5%. The above data shows that the proposed location information profile generation algorithm can efficiently generate the position information profile of each moving object, and the location profile information has high data availability. We made further time distribution statistics of the location points in the information profile, namely, the statistical number of location points over 24 hours, and the results are shown in Fig 8. There are different resident position points in different periods. Due to the particular features of the taxi, the number distribution of the location information profile is more uniform during those periods without the zero point. Thus, it is feasible that the location information profile generation algorithm establishes the profile data sets to recommend the LBS services for the LBS service requester during different periods in this paper. The algorithm can reduce the frequency of access to the LBS server and the exposure risk of the location information.

**Fig 9:**
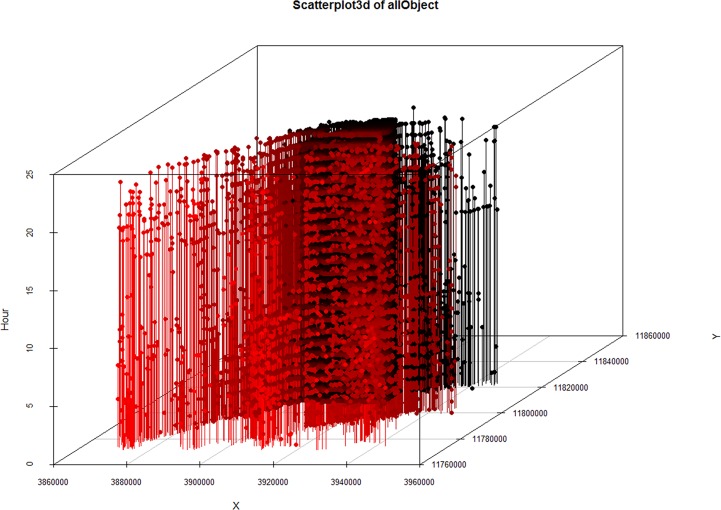
3Dscatterplot of the all object positions.

Finally, the 3D scatterplot of the object profile data (*x*, *y*) and the time (hour) is shown in Fig 9.

### Performance of service requests and the response processing algorithm

When the user requests the LBS service, the user's location generalization parameter (*r*) has a large influence on the user's location privacy protection level and the service recommendation results. In the experimental section, the generalization parameter *r* is set to be 100, 200, and 300 meters, and the corresponding service response to a user's location information profile is generalized as 100, 200, 300 meters; next, we conducted three groups of experiments according to the different values of *r*. We randomly selected 100 user position points as LBS service requesters from the data set in each group experiment. To illustrate expediently, we simplified the service request content theme as "in the next hour, where will I go?" while combining the characteristics of the data set in the experimental part. It must be explained that the service subject and the generalized parameter are set efficiently according to the actual situation in the practical application.

**Fig 10:**
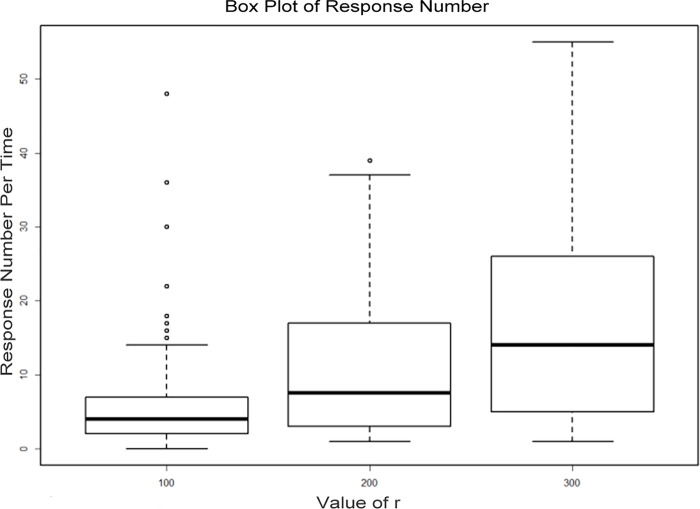
Number distribution of responsive users.

In the experimental part, according to the algorithm proposed in this paper, the LBS service request and response process are as follows: when a user requests location-based services, the user sets up the generalization parameter r and broadcasts the service request. We selected the moving objects within 100 meters away from the user in 5 minutes after sending the service request, and then, the selected object calculated its own location information profile according to the generalization parameter r; last, the object saw the location profile overlap of the object and responded to the request to recommend the result set if there is overlap. We set the parameter *r* at 100, 200 and 300 meters in the three groups of experiments, and the responsive user boxplot of 100 random service requests is shown in Fig 10.

When the r value is 100, 200, and 300 meters, the specific statistic of the responsive users (*RU*) to a number of users is shown in Table 2.

**Table 2:** Specific statistics of responsive users.

*r*	Minimum of *RU*	Maximum of *RU*	Average of *RU*	Failure ratio
100	0	48	6.01	1%
200	1	39	11.29	0%
300	1	55	17.55	0%

**Fig 11:**
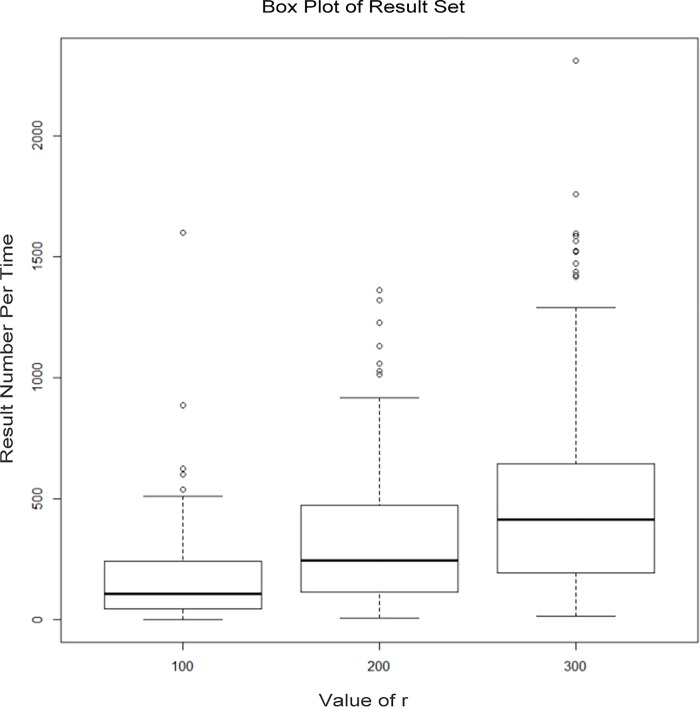
Number of result sets given by responsive users.

When service requests appear, the responsive user checks its location information profile and then sends the recommendation that meets the requirement to the service requesters, and the service requesters will obtain a location service alternative set. In the experiment, we counted the number of elements in the location service alternative set for each request with the *r* values of 100, 200, and 300 meters. The results are shown in Fig 11.

When the *r* value is 100, 200, and 300, the exact values of the result sets (*RS*) given by responsive users are shown in Table 3.

**Table 3:** Values of service result sets.

*r*	Minimum of *RS*	Maximum of *RS*	Average of *RS*	Failure ratio
100	0	1601	187.39	1%
200	6	1363	356.55	0%
300	12	2311	545.89	0%

It is not difficult to see from the above data that the number of responsive users and the service results increase with the increase in the *r* value. In the data set shown in this paper, when the *r* value is 100 meters, there is no user response. When the *r* value is 200 or 300 meters, the number of responsive users and the results of the recommended services are better. It could be that the number of responsive users is small because of the randomness of the requester positions. Then, the users might not receive a high quality location service, and this paper proposed the scheme of *k*-anonymity [5] as a supplement for this situation.

### Comparison among DCRLS, P2P and MobiCrowd

In this experiment, we compare execution efficiency and communication cost of the DCRLS, P2P and MobiCrowd. Although these three schemes all adopt the distributed system structure, but the dependency upon the untrusted third-party server of these three schemes are different. It is shown in Table 4. P2P uses neighboring user nodes to structure the k- anonymity data set and each location service need to access the server. MobiCrowd uses the model of virus transmission to provide location service, so it more depends on server and faces cold start. However, the DCRLS that proposed in our paper accesses the server when users cannot get location service recommendation, so it less depend on server.

**Table 4:** Framework comparison.

	DCRLS	P2P	MobiCrowd
Architecture tiers	2 tiers	2 tiers	3 tiers
Dependence on trusted third party	Low	heavy	medium
Privacy protect among peers	good	low	weak

**Fig 12:**
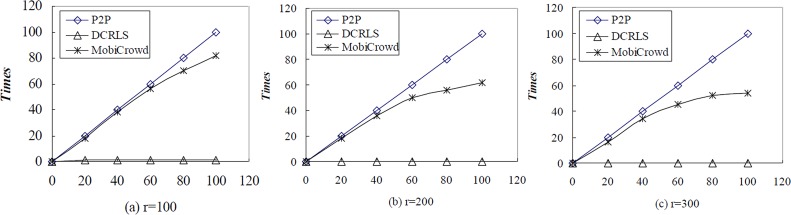
Times of access server comparison among P2P, DCRLS and MobiCrowd.

For the simplicity of comparison, we set parameter r as 100, 200, 300 meters and set parameter k in P2P algorithm as 10. These three algorithms request randomly 100 times location service respectively, the times change of access to server is shown in Fig 12.

In Fig 12, x-axis represents request times, y-axis represents access server times. When r sets 100, 200, 300 meters, times of access third-party server is great different among these three algorithms. And in P2P, every location service request needs to access server. But in DCRLS and MobiCrowd, with the increase of r and neighboring users number, the dependency upon location service reduces a lot. And in MobiCrowd, initial service needs to access server; the times of access server begin to decrease until it accumulates enough service result set. The DCRLS almost does not need access server, so it avoids privacy leakage because of the third-party server.

**Fig 13:**
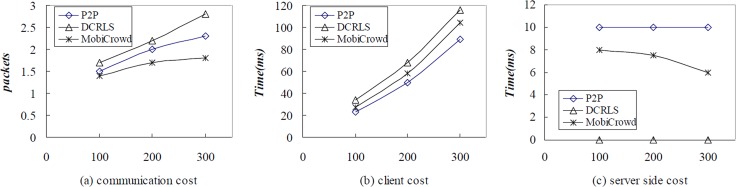
Performance comparison among P2P,DCRLS and MobiCrowd with r = 100,200,300.

We analyzed the average of communication cost when r is set 100, 200, 300 meters with TCP/IP data package. Besides, we compared the average of communication cost between server and client. The results are shown in Fig 13.

As shown in the Fig 13(A), the communication cost of these three algorithms is growing with the increase of r. The communication cost in the DCRLS is higher than P2P and MobiCrowd, because data transmission concentrates on the communication between client and neighboring users. And the communication cost is little lower in the MobiCrowd, because it adopts buffer mechanism. The Fig 13(B) and 13(C) show the time cost of server and client in these three algorithms. The time cost on server almost is invariant in P2P, because each service needs access server and k is set 10 in the experiment. The time cost on server is lower with the increase of communication range, user and chance that users get sharing service in MobiCrowd. And in DCRLS, the time cost on client is much than the other algorithms, but the time cost on server is low enough to ignore. It meets our requirements, reducing the times of access third-party server, to reduce the privacy leakage.

## Conclusions

For the location service privacy leak problems in a centralized system structure, this paper proposed a new distributed collaborative recommendation location service strategy. When the user requests location service, this strategy uses location information profile to recommend location service. It uses generalization and encryption to protect users’ location information. At last, we use real data set to do theoretical and experimental analysis for the algorithm in this strategy. The analysis shows that when a service request appears, the DCRLS can provide a sufficient number of service responsive users and a high quality of service request result sets. The DCRLS reduces the frequency of users’ access to the LBS server. And it overcomes the communication bottleneck and the defects that are easily attacked in the centralized systems structure and ensures the privacy safety. In future work, we will continue to perfect this strategy and improve the quality of the recommended LBS services by increasing the social relations in the mechanism that matches neighboring users and design a better method to protect location information.

## Supporting Information

S1 File Cab mobility traces data set. (RAR)

S2 File The minimal data set. (RAR)

S3 File The statement of cab mobility traces data set. (TXT)
